# Corrente de Sobrevivência à COVID-19

**DOI:** 10.36660/abc.20201171

**Published:** 2021-02-19

**Authors:** Sergio Timerman, Helio Penna Guimarães, Carlos Eduardo Rochitte, Thatiane Facholi Polastri, Marcelo Antônio Cartaxo Queiroga Lopes

**Affiliations:** 1 Universidade de São PauloInstituto do CoraçãoSão PauloSPBrasilUniversidade de São Paulo Instituto do Coração, São Paulo, SP - Brasil; 2 Hospital Israelita Albert EinsteinSão PauloSPBrasilHospital Israelita Albert Einstein, São Paulo, SP - Brasil; 3 Hospital do CoraçãoSão PauloSPBrasilHospital do Coração, São Paulo, SP - Brasil; 4 Hospital Alberto Urquiza WanderleyJoão PessoaPBBrasilHospital Alberto Urquiza Wanderley - Hemodinâmica e Cardiologia Intervencionista, João Pessoa, PB - Brasil; 5 Hospital Metropolitano Dom José Maria PiresJoão PessoaPBBrasilHospital Metropolitano Dom José Maria Pires, João Pessoa, PB - Brasil; 6 Sociedade Brasileira de CardiologiaRio de JaneiroRJBrasilSociedade Brasileira de Cardiologia, Rio de Janeiro, RJ - Brasil

**Keywords:** COVID-19, Coronavírus, Betacoronavírus, Medicina Baseada em Evidência, Sobrevivência, Cuidados Médicos

## Sumário

O termo Corrente de Sobrevivência fornece uma metáfora útil para os elementos do conceito de atendimento às vítimas da COVID 19. Seus cinco elos são:

-  CIÊNCIA – Medicina baseada em evidências (políticas públicas e políticas)
- SENSIBILIZAÇÃO – Sensibilização e conscientização da população
- TREINAMENTO – Treinamento de profissionais de saúde individualmente e em equipe
- ESTRUTURA – Equipamento e estruturação do pré-hospitalar e do hospital no atendimento à COVID-19
- RETOMADA – Retorno do paciente e da equipe

Uma forte Corrente de Sobrevivência pode melhorar as chances de sobrevivência e recuperação para as vítimas da COVID-19.

Realização: Sociedade Brasileira de Cardiologia (SBC), Associação Brasileira de Medicina de Emergência (ABRAMEDE)

Nota: Essa corrente se presta a informar e não a substituir o julgamento clínico do médico que, em última análise, deve determinar o tratamento apropriado para seus pacientes.

## Introdução

Em dezembro de 2019, a Comissão Municipal de Saúde e Saneamento de Wuhan, Província de Hubei, China, relatou um grupo de 27 casos de pneumonia de etiologia desconhecida, sendo 7 graves. Em janeiro de 2020, as autoridades chinesas identificaram um novo vírus da família *Coronaviridae*, então denominado ‘novo coronavírus’ ou 2019-nCoV e, posteriormente, SARS-CoV-2 (síndrome respiratória aguda grave coronavírus 2). A doença do novo coronavírus foi denominada COVID-19.^1^ Desde então, a COVID-19 alastrou-se, tendo acometido, até a primeira quinzena de outubro, 35.628.628 indivíduos em todo o mundo, sendo os Estados Unidos e o Brasil o epicentro, com 9.385.506 e 5.566.049 casos, respectivamente. No mesmo período, foram registrados 1.215.756 óbitos e o Brasil permanecia em segundo lugar nesse *ranking*, com 160.496 mortes.^2^

As medidas de emergência para tratar pacientes com COVID-19 e conter o surto são a principal prioridade em cada um dos países. No entanto, é provável que essas medidas resultem em danos colaterais para pacientes com outras doenças agudas, além de agravar as condições econômicas e sociais da população.

A Corrente de Sobrevivência à COVID-19 refere-se à cadeia de eventos que deve ocorrer em rápida sucessão para maximizar as chances de sobrevivência à doença e de restabelecimento de fluxos de saúde e sociais. Trata-se de uma metáfora simples para demonstrar ao público seu papel vital no controle da COVID-19, bem como o papel dos profissionais da saúde nessa corrente. Sugere que cada elo seja crítico e interdependente e que a corrente de sobrevivência seja forte quando consolidada em todos os seus elos, cuja abordagem efetiva possa ajudar a salvar vidas.

### Princípios gerais

Aprendendo com o drama mundial da COVID-19, este artigo aborda os principais aspectos do papel dos técnicos de saúde que, em paralelo com o corpo gerencial e médico, têm respondido às necessidades em constante mudança, proporcionando ambientes de cuidado adequado para os infectados e proteção para outros pacientes e operadores.

Esta explosão pandêmica, de fato, destacou a importância dos sistemas de saúde e hospitais em primeiro lugar e sua gestão constante como parte relevante de toda a governança neste momento dramático e complexo.

Este artigo tem por objetivo criar e descrever detalhadamente os componentes estruturais de uma Corrente de Sobrevivência à COVID-19. As recomendações contidas neste documento são baseadas nas evidências disponíveis no momento da sua elaboração e na opinião de especialistas. O conhecimento em relação à COVID-19 evolui de forma dinâmica e rápida; logo, os protocolos para reintrodução com segurança de atendimentos médicos e de procedimentos invasivos e não invasivos estão em constante evolução e adaptação. Este projeto foi idealizado pela Sociedade Brasileira de Cardiologia e pela Associação Brasileira de Medicina de Emergência, como uma fonte de referência para seus associados. As recomendações apresentadas, contudo, não devem ser usadas como única base para a definição de protocolos locais, devendo outras fontes atualizadas ser consideradas à medida que o conhecimento na área evolui.

O mesmo esforço sistemático, organizado e coordenado em uma comunidade continua sendo a recomendação mais forte que podemos fazer para salvar mais pessoas acometidas pela COVID-19. A metáfora dos elos de uma corrente provou ser bem-sucedida em muitos aspectos na ressuscitação cardiopulmonar.^3,4^ A utilização de uma Corrente de Sobrevivência à COVID-19 pode identificar pontos fracos nos ‘elos do sistema de atenção e combate à pandemia e assim auxiliar as comunidades a otimizarem o tratamento de pacientes críticos acometidos pela COVID-19.

Nesse contexto, todos passam a ser fundamentais e devem trabalhar em harmonia. Principais gestores de saúde, equipes de profissionais de saúde, do serviço técnico, da engenharia clínica, da gestão de risco, da farmácia, entre outros, constituem um sistema, cujo trabalho, à semelhança daquele de uma orquestra, requer gerenciamento dinâmico e operação em campo.

**Figura 1:**
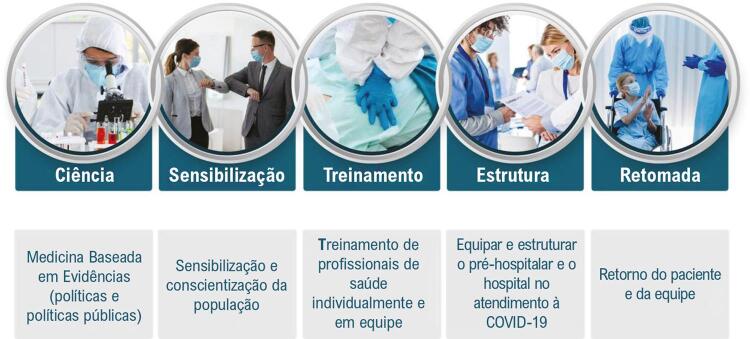
– Corrente de Sobrevivência à COVID-19.

A sobrevivência à forma grave da COVID-19 depende de uma sequência de intervenções críticas. Se uma dessas ações críticas for negligenciada ou atrasada, diminuem as chances de sobrevivência. Usamos o termo Corrente de Sobrevivência para descrever essa sequência. A Corrente de Sobrevivência à COVID-19 possui cinco elos interdependentes, como mostrado na Figura 1.

#### • CIÊNCIA: Medicina baseada em evidências (políticas e políticas públicas) – A cadeia de comando no sistema de saúde e o desafio COVID-19

Um ponto essencial é ocupar espaço no debate público, aproveitando o que a pandemia nos ensina como ocasião para uma avaliação objetiva sobre a governança dos sistemas de saúde. A discussão necessariamente continuará, como deve ser em uma democracia, mas é fundamental que as lições do período dramático sejam aprendidas.

A medicina baseada em evidências consiste no uso explícito e consciencioso das melhores evidências científicas para tomada de decisão no cuidado ao paciente. A medicina baseada em evidências busca unir a experiência do médico, os valores e desejos dos pacientes e a melhor evidência científica disponível.^5-7^ A pandemia da COVID-19 tem promovido sensíveis repercussões pessoais e políticas no âmbito da sociedade, impactando discussões calorosas e verdadeiros embates no agonismo e antagonismo de novos/velhos fármacos ou tratamentos. Esses cenários, no entanto, jamais modificam a demanda por melhores evidências de ensaios clínicos randomizados especificamente desenhados para determinar as modalidades de tratamento baseadas em evidências para reduzir a propagação dessa doença e prevenir o fardo de surtos futuros.^5-7^

#### • SENSIBILIZAÇÃO: Sensibilizar e conscientizar a população

Embora muitas terapias tenham sido sugeridas, até o momento nenhuma opção específica é capaz de tratar a COVID-19 ou agir de forma profilática quanto à infecção por SARS-CoV-2. A única intervenção atualmente viável e comprovada para reduzir a taxa de contágio parece ser o uso de medidas estritas de isolamento social para a população em geral.^5^ Os resultados de revisões sistemáticas e meta-análises apoiam o distanciamento físico de pelo menos 1 metro e fornecem estimativas quantitativas para modelos de rastreamento de contatos. O uso ideal de máscaras faciais, a higienização adequada das mãos e a proteção de contato em face/olhos nos ambientes públicos parecem promover impacto.^5,6^ MacIntyre et al.,^8^ em estudo randomizado comparando o uso de máscaras de tecido com máscaras cirúrgicas em profissionais de saúde, verificaram um número significativamente maior de acometimento respiratório entre os usuários de máscaras de tecido. Estudos e recomendações poster0iores,^8-12^ envolvendo simulação aplicada à distribuição populacional e transmissão da COVID-19, demonstraram que o uso contínuo de máscara facial (filtrante de 20-50% do ar exalado) pela população, mesmo assintomática, reduz significativamente a disseminação da COVID-19, com efeitos benéficos independentemente de potenciais grupos da população associados a maior risco. Tal redução pode ser potencialmente otimizada quando combinada ao distanciamento social. Portanto, é plausível e necessário considerar que todos os indivíduos utilizem máscaras quando expostos a aglomerações e outras situações de alto risco, em especial devido à maior transmissibilidade no período precoce assintomático da doença.

#### • TREINAMENTO: Treinamento dos profissionais de saúde (individual e em equipe)

A interrupção da educação médica permanente estendeu-se muito além de residência médica. O cancelamento de congressos, cursos e simpósios, a redução de equipes clínicas pelo afastamento de profissionais contaminados, a relutância quanto ao deslocamento para treinamentos, os confinamentos, o intenso acúmulo de funções e o aumento da carga de trabalho na pandemia da COVID-19 exigiram a implementação de tecnologias, assim como a adaptação e a ação imediata para minimizar a lacuna educacional.^13,14^

As teleconferências já haviam sido introduzidas como meio útil de educação continuada muito antes da COVID-19. No entanto, com a pandemia, o uso proeminente dessa tecnologia envolveu estratégias de ensino entre instituições,^13-18^ tornando-se o meio fundamental para a continuidade da educação clínica permanente e provando sua utilidade. Aplicativos diversos e multimídias de reunião virtual permitiram que departamentos clínicos implementassem palestras e sessões clínicas, bem como faculdades de medicina continuassem suas atividades, além de permitir que hospitais/diretorias clínicas continuassem a realizar seus relatórios e discussões de casos.

O ensino médico baseado em simulação manteve-se como estratégia adequada durante a pandemia, com adequações tais quais a redução do número de participantes, implementação de equipamentos de proteção individual e desinfecção de manequins.^19-22^

#### • ESTRUTURA: Equipamento e estruturação do pré-hospitalar e do hospital no atendimento à COVID-19

Diante da necessidade imediata de lidar com uma doença nova, a telemedicina propiciou aprendizado interdisciplinar e assistência a distância.^23^ Por exemplo, no Brasil, médicos intensivistas e emergencistas puderam atuar em consultorias para o atendimento de pacientes com COVID-19, contribuindo indiretamente para o manejo clínico baseado em evidências.^24-26^ O projeto Tele UTI, executado de forma colaborativa por cinco hospitais privados filantrópicos para atendimento de 2.500 leitos de unidade de terapia intensiva do Sistema Único de Saúde liderado pelo Hospital Israelita Albert Einstein, consiste em visita médica diária aos pacientes internados nas unidades de terapia intensiva, com ênfase nos casos de síndrome respiratória aguda grave e suspeita de COVID-19.^27^

#### • RETOMADA: Retorno do paciente e da equipe

O momento da reintrodução dos atendimentos médicos rotineiros e da vida em sociedade deve estar alinhado a políticas sociais e seguir as recomendações das autoridades competentes. É essencial que a comunidade médica e a sociedade permaneçam vigilantes e atentas a novas evidências e possíveis novos surtos.^26^

As seguintes medidas são indispensáveis: adequação da estrutura física com o objetivo de garantir distanciamento físico, através de demarcações sinalizadas no chão associadas à utilização de barreiras físicas no ambiente, como painéis de acrílico ou vidro; disponibilização do álcool em gel; sinalizações, como cartazes, placas e pôsteres, em locais estratégicos com informações sobre higienização das mãos, etiqueta da tosse e principais sinais e sintomas da COVID-19;^26-29^ e, fundamentalmente, o retorno da economia e a adequação social ao ‘novo hoje’.

## Rumo ao pós-COVID-19: Lições e desafios para hospitais e infraestruturas de saúde

Há evidências de possíveis repetições de ataques virais em um futuro próximo. A prevenção e a preparação são essenciais, especialmente para o setor da saúde.

## Conclusão

A primeira lição é que este dramático período que vivemos exige coragem para mudar: quem trabalha na área da saúde tem que repensar os modelos arquitetônicos. Os técnicos fizeram milagres ao adaptar os hospitais atuais para atender drasticamente às novas necessidades e, no futuro, devem ser incluídos nos processos de planejamento e *design*. A governança dos sistemas de saúde tem que considerar a necessidade de menos fragmentação, de forte coordenação nacional. Há que se levar em conta que neste momento temos que caminhar de forma sustentável, tendo como metas a prevenção e a preparação, além de um desenvolvimento econômico focado no respeito às pessoas, à comunidade e ao meio ambiente. Assim, devemos nos lembrar da fala de Maquiavel e aproveitar uma crise dramática.

O conceito da Corrente de Sobrevivência à COVID-19 ressalta vários princípios importantes e, se qualquer elo da cadeia for inadequado, as taxas de sobrevivência serão baixas. A fraqueza nos componentes do sistema é a principal explicação para a variabilidade nas taxas de sobrevivência.

Embora todos os elos devam ser fortes, sempre surge a pergunta inevitável: qual o mais importante? Certamente o reconhecimento da emergência e o início da cadeia são essenciais e, se ninguém reconhecer a emergência e começar a agir, a sobrevivência será baixa.

Como a ‘Estrutura’ é a única intervenção ‘suficiente’, ou seja, é o elo que trata a COVID-19, é frequentemente proclamado como ‘o fator mais importante na determinação da sobrevida’. A eficácia não pode ser identificada examinando um elo individual, pois todo o sistema deve ser avaliado. A verdade, no entanto, é ainda mais satisfatória e mais condizente com o conceito de Corrente de Sobrevivência, em que cada elo é importante.

Uma forte Corrente de Sobrevivência pode melhorar as chances de sobrevivência e recuperação das vítimas de COVID-19.
