# Supplementary material for: Corrente de Sobrevivência à COVID-19
Source: Arq Bras Cardiol. 2021 Feb 19;116(2):351–4. [Article in Portuguese] doi: 10.36660/abc.20201171 (PMC7909961; doi:10.36660/abc.20201171)
Supplement: Translation

# Translation: COVID-19 Chain of Survival 2020

**Authors:** Sergio Timerman, Helio Penna Guimarães, Carlos Eduardo Rochitte, Thatiane Facholi Polastri, Marcelo Antônio Cartaxo Queiroga Lopes

**Affiliations:** 1 BrasilUniversidade de São Paulo Instituto do Coração, São Paulo, SP - Brasil; 2 BrasilHospital Israelita Albert Einstein, São Paulo, SP - Brasil; 3 BrasilHospital do Coração, São Paulo, SP - Brasil; 4 BrasilHospital Alberto Urquiza Wanderley - Hemodinâmica e Cardiologia Intervencionista, João Pessoa, PB - Brasil; 5 BrasilHospital Metropolitano Dom José Maria Pires, João Pessoa, PB - Brasil; 6 BrasilSociedade Brasileira de Cardiologia, Rio de Janeiro, RJ - Brasil

**Keywords:** COVID-19, Coronavirus, Betacoronavirus, Evidence Based Medicine, Survival, Medical Care

## Abstract 

The term Chain of Survival provides a useful metaphor for the elements involved in the COVID-19 management. The 5 links of the COVID-19 Chain of Survival are:

-  SCIENCE - Evidence-based medicine (public policies and policies)
-  AWARENESS - Increasing population sensitization and awareness
-  TRAINING - Individual or team training for healthcare professionals
-  STRUCTURE - Equip and structure the pre-hospital and in-hospital phases of COVID-19 management
-  RETURN - Return of patients and healthcare professionals

A strong Chain of Survival can improve the chances of survival and recovery for victims of COVID-19.

Initiative: Brazilian Society of Cardiology (SBC) and Brazilian Association of Emergency Medicine (ABRAMEDE).

Note: The COVID-19 Chain of Survival is aimed at providing information and not at replacing the clinical judgement of physicians, who should determine the appropriate treatment for their patients.

## Introduction

In December 2019, the Municipal Health and Sanitation Commission of Wuhan, Hubei Province, China, reported a group of 27 cases of pneumonia of unknown etiology, 7 of which were severe. In January 2020, the Chinese authorities identified a new virus in the *Coronaviridae* family that has been named ‘new coronavirus’, 2019-nCoV, and, subsequently, SARS-CoV-2 (severe acute respiratory syndrome coronavirus 2). The disease associated with the new coronavirus has been called COVID-19.^1^ Since then, COVID-19 has spread, affecting 35 628 628 individuals worldwide by the first fifteen days of October, having the United States and Brazil as its epicenter, with 9 385 506 and 5 566 049 cases, respectively. Regarding the number of deaths in the same period, 1 215 756, Brazil ranks second, with 160 496 deaths.^2^

The emergency measures to treat patients with COVID-19 and restrain the outbreak are number one priority in those countries. However, those measures might result in collateral damage for patients with other acute diseases, in addition to worsening the socioeconomic conditions in those countries.

The Chain of Survival refers to a chain of events that should occur in rapid sequence to maximize the chances of surviving COVID-19 and restoring the health and social flows. It is a simple metaphor to demonstrate the population’s vital role in controlling COVID-19, in addition to the role played by healthcare professionals in that control. It suggests that each link of the chain is critical and depends on the preceding one, and that the chain of survival is not only strong when all its links are consolidated, but it might help save lives through the effective approach to its links.

### General principles

This article addresses the major challenges faced by healthcare technicians, who, with health managers and physicians, have responded to the ever-changing needs regarding COVID-19 management, providing adequate healthcare environments to infected individuals as well as protection to other patients and care providers.

This pandemic has highlighted the importance of the healthcare systems and hospitals, as well as the need for their constant management as a major part of governance in this challenging and complex situation.

This article aims at creating and describing the structural components of a COVID-19 Chain of Survival. The recommendations in this document are based on evidence available at the time of its elaboration and on expert opinions. Because the knowledge about COVID-19 rapidly evolves, the protocols for the safe return of medical care and invasive and non-invasive procedures are constantly evolving and adapting. This project has been idealized by the Brazilian Society of Cardiology and the Brazilian Association of Emergency Medicine (ABRAMEDE) to serve as reference for their associates. The recommendations presented, however, should not be used as the sole base to define local protocols; other updated sources should be considered as knowledge in the field evolves.

Systematic, organized and coordinated effort in the community continues to be the most powerful recommendation we can make to save individuals affected by COVID-19. The metaphor of the links in a chain has proved to be successful in many aspects regarding cardiopulmonary resuscitation.^3,4^ The use of the COVID-19 Chain of Survival can identify weak spots not only in the healthcare system ‘links’ but also in the fight against the pandemic, and, thus, contribute to optimize the treatment of critical patients with COVID-19. Therefore, all individuals involved are important and should work in harmony. Major health managers, healthcare professionals, technicians, clinical engineers, risk managers and pharmacists, among others, should work as in an orchestra, with dynamic system management.

Surviving severe COVID-19 depends on a sequence of critical interventions. If one of such critical interventions is neglected or delayed, the chances of survival decrease. The term ‘Chain of Survival’ is used to describe that sequence. Figure 1 shows the 5 interdependent links in the COVID-19 Chain of Survival.

**Figure 1:** – COVID-19 Chain of Survival.

#### • SCIENCE: Evidence-based medicine (policies and public policies) – The chain of command in the health system and the COVID-19 challenge

An essential point is to participate in the public debate, learning with the pandemic and objectively assessing the governance of health systems. The discussion will necessarily continue, as it should in a democracy, but it is paramount to learn the lessons of this challenging period.

Evidence-based medicine is the explicit and conscientious use of the best scientific evidence for the decision-making regarding patient care. Evidence-based medicine aims at uniting the physician’s experience, the patient values and preferences, and the best scientific evidence available.^5-7^ The COVID-19 pandemic has had personal and political repercussions, yielding impassioned discussions and true clashes in the agonism and antagonism of new/old drugs or treatments. This scenario, however, does not change the demand for better evidence deriving from randomized clinical trials specifically designed to determine the evidence-based modalities of treatment to reduce the COVID-19 spread and prevent the burden of future outbreaks.^5-7^

#### • AWARENESS: Increasing population sensitization and awareness

Although several therapies have been suggested, no specific option has been able to successfully treat COVID-19 or prevent SARS-CoV-2 infection so far. The only feasible intervention that has proven to reduce the transmission rate seems to be the use of strict social distancing measures for the general population.^5^ The results of systematic reviews and metanalyses have supported the physical distancing of at least 1 meter and have provided quantitative estimates for contact tracing models. The ideal use of face masks, proper hand hygiene, and face/eye protection against contact in public places seem to have an impact.^5,6^ MacIntyre et al.,^8^ in a randomized study comparing the use of cloth masks and medical masks in healthcare workers, have found a significantly higher rate of respiratory infection among cloth masks users. Other studies and recommendations,^8-12^ involving simulation applied to population distribution and COVID-19 transmission, have shown that the continuous use of face masks (filtering 20-50% of exhaled air) by the general population, even when asymptomatic, significantly reduces COVID-19 spread, with beneficial effects regardless of the population groups associated with higher risk. That reduction can be potentially optimized when social distancing is associated. Therefore, it is plausible and necessary to consider that all individuals should wear masks when exposed to agglomerations and other high-risk situations, especially because of the higher transmissibility in the early asymptomatic phase of COVID-19.

#### • TRAINING: Individual and team training for healthcare professionals

The interruption of permanent medical education goes beyond medical residence. The cancellation of medical congresses, courses and symposia, the reduction in clinical teams due to absence of contaminated professionals, the reluctance to attend trainings, the confinements, the taking on colleague’s tasks, and the increase in the workload during the COVID-19 pandemic have required the implementation of technologies, as well as adjustments and immediate action to minimize the educational gap.^13,14^

Teleconferences had already been introduced as a useful tool for continuing education long before COVID-19 appearance. With the pandemic, their prominent use involved in exchanging learning strategies between institutions^13-18^ made teleconferences the fundamental mean to provide permanent clinical education, proving their usefulness. Several apps and multimedia conferencing have allowed not only clinical departments to implement lectures and clinical sessions, but also medical schools to continue their activities. In addition, hospitals and clinical units could continue to issue reports and discuss their cases.

Simulation-based medical education has been an adequate tool for learning during the COVID-19 pandemic, provided some adjustments were made, such as the reduction in the number of participants, use of personal protection equipment, and disinfection of mannequins.^19-22^

#### • STRUCTURE: Equip and structure the pre-hospital and in-hospital phases of COVID-19 management

In face of the immediate need to manage a novel disease, telemedicine has enabled interdisciplinary and distance learning.^23^ In Brazil, intensive care and emergency physicians could provide remote care to patients with COVID-19, indirectly contributing to evidence-based clinical management.^24-26^ The Tele UTI project is a collaborative initiative of five private philanthropic hospitals to provide care to 2500 intensive care beds from the Brazilian Unified Public Health System, under the leadership of the Hospital Israelita Albert Einstein. The project consists in daily remote medical visits to intensive care patients, with emphasis on severe acute respiratory syndrome and suspected COVID-19 cases.^27^

#### • RETURN: Return of patients and healthcare professionals

The return of routine medical visits and social life should be aligned with social policies and follow the recommendations of competent authorities. It is mandatory that the medical community and the society remain vigilant and pay careful attention to new evidence and possible new outbreaks.^26^

The following measures are imperative in healthcare settings: proper physical structure to ensure physical distancing by using floor signaling in association with physical barriers, such as acrylic or glass panels; alcohol gel provision; banners and posters displayed at strategic places with information on hand hygiene, cough etiquette, and COVID-19 major signs and symptoms.^26-29^ Last but not least, the economy recovery and the social adequacy to the ‘new today’ are fundamental.

Towards post-COVID-19: Lessons and challenges to hospitals and health infrastructure

There is evidence of possible repetitions of viral attacks soon. Prevention and preparedness are essential, especially for the healthcare sector.

## Conclusion

The first lesson to be learned is that this challenging period requires courage to change: those who work in the healthcare sector must rethink the architectonic models. Technicians have made miracles by adapting the current hospitals to promptly meet the new demand and should be included in future planning and design processes. The governance of the health systems must consider the need for less fragmentation and stronger national coordination. It is worth considering that we currently must be even more sustainable, aiming at prevention and preparedness, with an economic development focused on respect to people, the community, and the environment. We should, thus, remember Machiavelli and take advantage from a dramatic crisis.

The concept of the COVID-19 Chain of Survival highlights several important principles, and any frail link in the chain reduces the survival rates. A weak component of the system is the major reason for variability in the survival rates.

Although all links should be strong, the unavoidable question persists: which is the most important? Recognizing the emergency and starting the chain are certainly essential; thus, if that does not occur, the survival decreases.

Because ‘Structure’ is the only ‘sufficient’ intervention, that is, the link that treats COVID-19, it is often referred as ‘the most important factor in determining survival’. However, the efficacy of the chain cannot be assessed based on only one individual link; the chain should be assessed as a whole. In fact, the truth is even more satisfactory and suitable for the concept of the Chain of Survival, in which each link matters.

A strong Chain of Survival can improve the chances of survival and recovery for COVID-19 victims.
